# Uncomplicated Malaria in Infancy and Neurodevelopment at 1 and 6 Years: Evidence From the MiPPAD Birth Cohort Study of Benin

**DOI:** 10.1111/tmi.70179

**Published:** 2026-06-19

**Authors:** Juthathip Khongpetch, Michael J. Boivin, Michel Cot, Jacqueline Milet, Roméo Zoumenou, Christophe Fermanian, Achille Massougbodji, Florence Bodeau‐Livinec

**Affiliations:** ^1^ Cancer Epidemiology Unit, Medical Science Department University of Turin and CPO Piemonte Turin Italy; ^2^ University School for Advanced Studies IUSS Pavia Pavia PV Italy; ^3^ Department of Psychiatry Michigan State University East Lansing Michigan USA; ^4^ UMR 261‐MERIT, Institut de Recherche pour le Développement (IRD), Université Paris Cite Paris France; ^5^ Institut de Recherche Clinique du Benin (IRCB) Abomey‐Calavi Benin; ^6^ Univ Rennes, EHESP, Inserm, IRSET (Institut de recherche en santé, environnement et travail) – UMR 1085, Arènes UMR 6051 Rennes France; ^7^ Univ Rennes, EHESP, Inserm, Irset (Institut de recherche en santé, environnement et travail) – UMR_S 1085 Rennes France

**Keywords:** anaemia, birth cohort study, neurodevelopment, Sub‐Saharan Africa, uncomplicated malaria

## Abstract

In malaria‐endemic regions, repeated episodes of uncomplicated malaria during infancy are common; however, their associations with neurodevelopment remain unclear. We examined relationships between malaria frequency in infancy and neurodevelopmental outcomes in early and middle childhood, using data from the APEC birth cohort nested within the MiPPAD birth cohort study of Benin. Children were enrolled between January 2010 and June 2011 and followed from birth to 24 months of age. Malaria was assessed at scheduled visits at 6, 9 and 12 months using both PCR and microscopy, and at unscheduled visits for symptomatic episodes using only microscopy; haemoglobin was measured concurrently. Neurodevelopment was assessed at age 1 and 6 years. Analyses included 311 children at 1‐year and 248 at 6‐year assessments. Malaria exposure was defined as the number of episodes from birth to 1‐year neurodevelopmental assessment for three measures: (i) total malaria (PCR‐ or microscopy‐positive), (ii) symptomatic malaria (positive with fever) and (iii) malaria with anaemia (positive with haemoglobin < 11.0 g/dL). Associations with neurodevelopment and haemoglobin's mediating role were assessed using adjusted generalized linear models and mediation analysis. We found that a higher number of symptomatic malaria episodes during infancy was associated with lower motor scores at age one (*β*: −2.81, [−4.73, −0.89]) and age six (*β*: −1.43, [−2.65, −0.22]), partially and potentially mediated by haemoglobin concentration. Malaria with anaemia was associated with lower motor scores at age one (*β*: −1.57, [−3.04, −0.10]) and age six (*β*: −0.96, [−1.86, −0.05]). No consistent associations were observed with cognitive outcomes. These findings indicate that uncomplicated malaria in infancy is an important risk factor for impaired child development and should not be overlooked. Integrating anaemia screening and management into malaria control and routine child health services may help mitigate long‐term developmental impacts in malaria‐endemic settings.

## 1 Introduction

In malaria‐endemic regions, children are frequently exposed to *Plasmodium* infections during infancy, a critical period for brain growth and neurodevelopment, especially for motor development. Disruptions during this period may have lasting effects on learning, attention and socioemotional functioning later in childhood [1, 2, 3].

Although malaria has been associated with impaired neurodevelopment in children, the existing evidence remains limited. Most studies have focused on prenatal malaria exposure or severe and complicated malaria during early childhood, often with short‐term follow‐up [4, 5, 6, 7, 8, 9]. In contrast, uncomplicated malaria, a common clinical presentation in endemic settings, has been understudied. Only a few cohort studies have examined its association with childhood neurodevelopment, with mixed findings and limited follow‐up beyond early childhood [5, 10].

Malaria may affect neurodevelopment through immune and inflammatory responses, malaria‐related anaemia and illness‐related reductions in children's activity [5, 11, 12, 13, 14]. Among these mechanisms, anaemia is a frequent clinical presentation in young children; however, its mediating role is poorly understood.

Using data from a birth cohort in southern Benin, we examined whether exposure to uncomplicated malaria during infancy is associated with neurodevelopmental outcomes at ages one and six, and whether anaemia mediates these associations. By focusing on the cumulative burden of uncomplicated malaria with long‐term follow‐up, this study addresses an important evidence gap on the neurodevelopmental consequences of early‐life malaria exposure in endemic settings.

## 2 Methods

### 2.1 Study Design

This study used data from the *Apport des Premiers Episodes de Paludisme Chez l'Enfant* (APEC) cohort, nested within the *Malaria in Pregnancy Preventive Alternative Drugs (MiPPAD) Birth Cohort study of Benin* [15, 16], which followed 1136 live singletons born to women enrolled in the MiPPAD clinical trial (NCT00811421). The trial was conducted between January 2010 and May 2012 in Benin to evaluate the effectiveness of sulphadoxine–pyrimethamine (SP) and mefloquine (MQ) for intermittent preventive treatment in pregnancy (IPTp).

A total of 1183 pregnant women were enrolled in the MiPPAD trial from three healthcare centres in Allada district (Sékou, Allada, Attogon). Eligible women were: (i) permanent residency in the study area; (ii) gestational age ≤ 28 weeks; (iii) HIV‐negative; (iv) no known allergy to SP or MQ; (v) no SP or MQ treatment within the previous 4 weeks; and (vi) no severe renal, hepatic, psychiatric, or neurological disorders. Of these, 1005 participated in an ancillary malaria risk study and gave birth to 862 live singletons, of whom the first 400 (200 born to anaemic mothers and 200 to non‐anaemic mothers) were enrolled in the APEC cohort between January 2010 and June 2011.

In the MiPPAD trial, maternal data were collected at enrolment, 1 month later and at delivery. Malaria was assessed by thick blood smear (TBS) only if participants had clinical symptoms during pregnancy, while at delivery, testing was done using TBS and PCR on peripheral venous blood, cord blood, placental biopsy and a placental smear was taken for histology. Haemoglobin concentration (Hb), temperature, age, literacy, gravidity, gestational age, weight, height and IPTp regimen (MQ full dose, MQ split dose, or SP) were also recorded. Unscheduled visits were conducted as needed from enrolment to 2 months after delivery, during which TBS and Hb were assessed if participants had malaria symptoms.

In the APEC cohort, infants were followed from birth to 24 months. At birth, sex, gestational age, length and birthweight were recorded. At scheduled visits at 6, 9 and 12 months, malaria tests were conducted (PCR and TBS) and Hb, temperature, weight and height were measured. Unscheduled visits were conducted as needed, during which TBS and Hb were measured if children had malaria symptoms. No scheduled visits occurred between 12 and 24 months.

At the 1‐year assessment, motor and cognitive functions were evaluated using Mullen Scales of Early Learning (MSEL) [17]. Data collected included weight, height, temperature, Hb (g/dL), malaria (PCR and TBS), age at neurodevelopmental assessment (month), maternal education, household possession score (range 0–15, based on electricity, television, toilet, refrigerator, livestock and vehicles) and the Home Observation for Measurement of the Environment inventory (HOME), derived from maternal interviews and observed mother–child interactions during home visit, with higher scores reflecting a more supportive learning environment [18].

At the 6‐year assessment, motor function was assessed using Bruininks‐Oseretsky Test of Motor Proficiency, Second Edition (BOT‐2) [19], and cognitive function using Kaufman Assessment Battery for Children, Second Edition (KABC‐II) [20]. Data collected included weight, height, temperature, Hb (g/dL), malaria (PCR and TBS) and age at neurodevelopmental assessment (month).

Malaria testing primarily targeted *Plasmodium falciparum*, the predominant species in southern Benin [21, 22]. Children with malaria symptoms and a positive TBS received free treatment and follow‐up care, and neurodevelopmental testing was postponed until recovery. No child was assessed during symptomatic malaria or other illness. Detailed descriptions of the MiPPAD and APEC cohorts have been published elsewhere [12, 22, 23]. Data collection procedures are summarized in Supporting Information S1.

### 2.2 Population Selection

We selected APEC children because they had scheduled visits included malaria testing by both PCR and TBS. To examine the association between early‐life malaria and neurodevelopmental outcomes at 1 year (1‐year analysis), we included children with non‐missing MSEL results. For association at 6 years (6‐year analysis), we included children with non‐missing BOT‐2 and KABC‐II results. In both analyses, the exposure window was defined from birth to the last visit before MSEL‐assessment to evaluate whether infancy malaria was associated with neurodevelopment in early and later childhood. Therefore, the 6‐year analysis was restricted to children with available neurodevelopmental data at both ages.

Population selection flowchart is shown in Figure 1. Of the 400 children enrolled, 76 were excluded before the 12‐month follow‐up due to death, migration, refusal, or other reasons. At 1 year, nine children were lost to follow‐up and four had incomplete results, yielding 311 children included in the analysis. At 6 years, 15 children had died, 40 were lost to follow‐up and eight had incomplete results, leaving 248 children included in the analysis.

**FIGURE 1:**
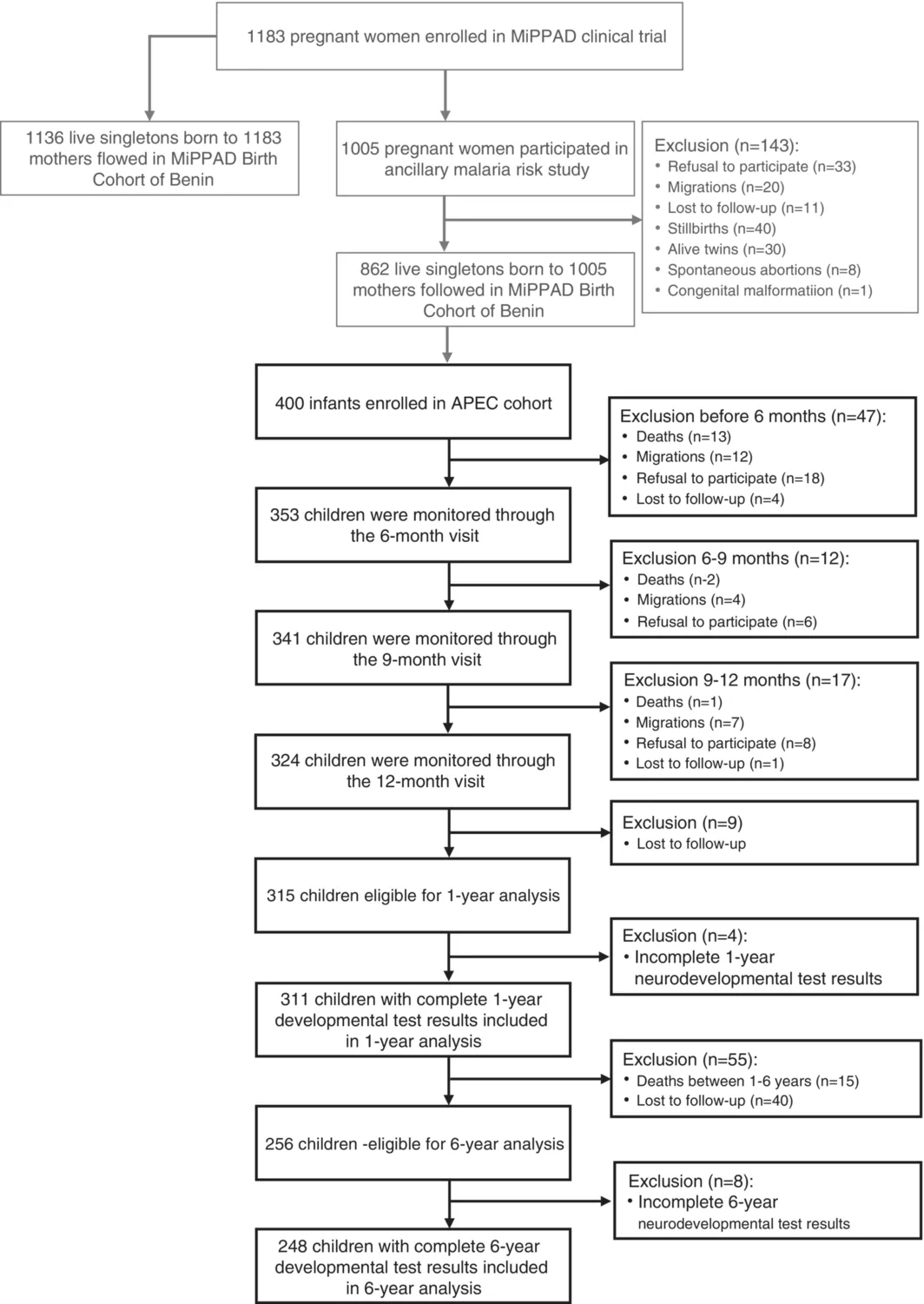
Population selection flowchart (adapted from Accrombessi et al. [12]). A total of 1136 live singleton children were followed in the MiPPAD Birth Cohort of Benin from birth to 12 months. For both the 1‐ and 6‐year analyses, malaria exposure was defined from birth until the date of 1‐year neurodevelopmental assessment, conducted at approximately 12 months of age (median age: 12 months, range: 12–16). Therefore, the 6‐year analysis was restricted to children with available neurodevelopmental data at both ages.

### 2.3 Assessment of Malaria Exposures

Malaria exposures from birth to the last visit before 1‐year neurodevelopmental assessment were defined as the number of episodes for three measures; (i) total malaria; PCR or TBS positive, to capture overall effects of both low‐ and high‐density infections, (ii) symptomatic malaria; PCR or TBS positive with an axillary temperature > 37.5°C at the visit or with a history of reported fever within the prior 24 h [24] and (iii) malaria with anaemia; PCR or TBS positive with Hb < 11.0 g/dL [25]. Malaria episodes were defined as positive tests occurring more than 14 days after a previous positive test; positives within 14 days were considered part of the same episode [5].

### 2.4 Assessment of Childhood Neurodevelopment

At age 12 months (median 12 months; range 12–16), neurodevelopment was assessed using the Mullen Scales of Early Learning (MSEL), a standardized tool used to assess cognitive and motor development in children up to 68 months and previously applied in this setting [17, 26, 27, 28]. The MSEL includes a Gross Motor scale (GM) and an Early Learning Composite (ELC), derived from visual reception, fine motor, receptive language and expressive language subscales. Raw subscale scores were converted into age‐standardized *T scores* using U.S. normative data and combined to derive the ELC score, predefined as the primary outcome. The MSEL was translated from English into French and backtranslated with approval from the publisher. Instructions were administered in the local language (Fon) by trained assessors following established procedures in this setting.

At age 6 years (median 72 months; range 70–110), neurodevelopment was assessed using BOT‐2 [19] and KABC‐II [20], standardized tools used to assess motor and cognitive performance at school age and previously applied in this setting [26]. The BOT‐2 measures motor proficiency in children aged 4–21 years and provides age‐ and sex‐standardized composite scores based on U.S. normative data. The KABC‐II assesses cognitive functions in children aged 3–18 years and was administered using the French version (normed on children in France). Instructions were delivered in the local language (Fon) by trained assessors following established procedures in this setting. The Mental Processing Index (MPI) and Non‐Verbal Index (NVI) were selected as primary cognitive outcomes. The validity of the KABC‐II in this setting has been examined in previous studies [26, 27, 29].

For all neurodevelopmental assessments, higher scores indicate better performance. As no local normative data were available, analyses were based on within‐cohort comparisons. Assessors were blinded to previous malaria exposure. Further details on these assessments are described elsewhere [26, 27, 28, 29].

### 2.5 Statistical Analysis

Descriptive statistics compared characteristics of mother–child pairs included and excluded from analyses. Bivariate associations between covariates and neurodevelopmental outcomes were examined using unadjusted generalized linear models (GLMs) with Gaussian distribution and identity link, performed on non‐imputed data. Missingness across covariates was < 3%. Height‐for‐age *z*‐score at 1 year (HAZ), household possession and HOME scores were mean‐imputed. No other variables had missing data.

Associations between early‐life malaria and neurodevelopment were assessed using GLMs adjusted for covariates selected through literature review, directed acyclic graph (DAG; Supporting Information S2) and bivariate screening (*p* < 0.2). Malaria exposures were modelled as continuous variables. Separate models were fitted for each exposure‐outcome pair at both ages. Final models included sex, maternal IPTp regimen, maternal malaria at delivery (PCR or TBS positive in maternal peripheral venous blood), primigravidity and maternal education as categorical variables and age at neurodevelopmental assessment, HAZ, HOME and household possession score as continuous variables. Age was added to all models to account for variability in testing time. MSEL scores were not included in 6‐year models to avoid overadjustment.

We tested whether Hb at the last visit before MSEL‐assessment (pre‐MSEL Hb) mediated the association between early‐life malaria and neurodevelopment at ages one and six. Following the causal mediation framework in the R package *mediation* [30], we specified two models: (i) a mediator model estimating the association between malaria and pre‐MSEL Hb, and (ii) an outcome model estimating the association between malaria and neurodevelopment, adjusting for pre‐MSEL Hb (average direct effect, ADE). The average causal mediation effect (ACME) was estimated using the *mediate* function with nonparametric bootstrapping based on 5000 simulations. A *p*‐value < 0.05 was considered statistically significant for ACME, indicating evidence of mediation.

Several sensitivity analyses were conducted. First, we additionally adjusted for Hb measured at the time of neurodevelopmental assessment to assess whether associations between early‐life malaria and neurodevelopment were independent of concurrent anaemia. Second, we assessed the mediating role of Hb using two alternative measures: (i) the average Hb from birth to the last visit before MSEL‐assessment, reflecting chronic anaemia, and (ii) the lowest recorded Hb during this period, reflecting anaemia severity. Third, we repeated analyses excluding PCR‐positive but TBS‐negative episodes to assess whether the associations were sensitive to differences in test sensitivity. Lastly, in the 6‐year analysis, we included malaria episodes occurring after MSEL‐assessment to 24 months to evaluate whether extending the exposure window changes estimated associations. All analyses were performed using R Software (version 4.5.2).

### 2.6 Ethics Statement

Ethical approval was obtained from the institutional review boards of the Beninese Ethical Committee of the Faculté des Sciences de la Santé (FSS), the Committee of Ethical Research of the Applied Biomedical Sciences Institute (CER‐ISBA) in Benin, New York University (IRB #09‐1253, USA) and the Research Institute for Development's (IRD) Consultative Ethics Committee in France. Written informed consent was obtained from all participants, with thumbprint consent for those unable to read or write.

## 3 Results

### 3.1 Study Population and Malaria Episodes

Characteristics of mother–child pairs in the 1‐ and 6‐year analyses are shown in Table 1. Included and excluded children were comparable across key characteristics. However, mothers of children included in the 6‐year analysis were slightly older. Most mothers were aged ≥ 25 years (1‐year: 58%; 6‐year: 62%) and multigravidae (1‐year: 84%; 6‐year: 86%). Approximately 40% had formal education, and the mean household possession score was 5.2 (SD 2.7). At delivery, around 30% of mothers were malaria positive. Among included children, 51% were female, 6% were born preterm and 7% had low birthweight.

**TABLE 1:** Descriptive characteristics of mother–child pairs included and excluded from the 1‐ and 6‐year analyses.

	Included 1‐year analysis (*N* = 311)	Excluded 1‐year analysis (*N* = 89)	Included 6‐year analysis (*N* = 248)	Excluded 6‐year analysis (*N* = 67)
Total malaria				
0 episode	155 (49.8)	—	125 (50.4)	33 (49.3)
1 episode	76 (24.4)	—	58 (23.4)	19 (28.4)
2 episodes	43 (13.8)	—	36 (14.5)	7 (10.4)
≥ 3 episodes	37 (11.9)	—	29 (11.7)	8 (11.9)
Symptomatic malaria				
0 episode	190 (61.1)	—	152 (61.3)	42 (62.7)
1 episode	78 (25.1)	—	60 (24.2)	18 (26.9)
2 episodes	34 (10.9)	—	30 (12.1)	4 (6.0)
≥ 3 episodes	9 (2.9)	—	6 (2.4)	3 (4.5)
Malaria with anaemia				
0 episode	177 (56.9)	—	140 (56.5)	40 (59.7)
1 episode	68 (21.9)	—	56 (22.6)	13 (19.4)
2 episodes	36 (11.6)	—	28 (11.3)	8 (11.9)
≥ 3 episodes	30 (9.6)	—	24 (9.7)	6 (9.0)
Sex				
Male	151 (48.6)	37 (41.6)	120 (48.4)	33 (49.3)
Female	160 (51.4)	52 (58.4)	128 (51.6)	34 (50.7)
Preterm birth				
No	292 (93.9)	84 (94.4)	235 (94.8)	61 (91.0)
Yes	19 (6.1)	5 (5.6)	13 (5.2)	6 (9.0)
Low birthweight				
No	285 (92.2)	77 (86.5)	228 (92.7)	61 (91.0)
Yes	24 (7.8)	12 (13.5)	18 (7.3)	6 (9.0)
HAZ				
< −2 (Stunting)	68 (22.1)	—	55 (22.5)	13 (20.0)
≥ −2	240 (77.9)	—	189 (77.5)	52 (80.0)
Hb at 1‐year assessment (g/dL)				
< 11.0 (anaemic)	197 (65)	—	—	—
≥ 11.0	106 (35)	—	—	—
Hb at 6‐year assessment (g/dL)				
< 11.0 (anaemic)	—	—	55 (26.3)	—
≥ 11.0	—	—	154 (73.7)	—
Pre‐MSEL Hb				
< 11.0 (anaemic)	200 (64.3)	—	161 (64.9)	41 (61.2)
≥ 11.0	111 (35.7)	—	87 (35.1)	26 (38.8)
Number of visits				
< 6 (median)	126 (40.5)	—	94 (37.9)	34 (50.7)*
≥ 6	185 (59.5)	—	154 (62.1)	33 (49.3)
Age at the last visit (month)				
< 12 (median)	39 (12.5)	76 (85.4)*	32 (12.9)	9 (13.4)
≥ 12	272 (87.5)	13 (14.6)	216 (87.1)	58 (86.6)
Mother's age (year)				
< 25	130 (41.8)	37 (41.6)	95 (38.3)	37 (55.2)*
≥ 25	181 (58.2)	52 (58.4)	153 (61.7)	30 (44.8)
Age at 1‐year assessment (month)				
< 13 (mean)	160 (51.4)	—	—	—
≥ 13	151 (48.6)	—	—	—
Age at 6‐year assessment (month)				
< 73 (median)	—	—	69 (27.8)	—
≥ 73	—	—	179 (72.2)	—
Maternal literacy				
No	238 (76.5)	77 (86.5)	194 (78.2)	48 (71.6)
Yes	73 (23.5)	12 (13.5)	54 (21.8)	19 (28.4)
Mother's education				
None	185 (59.5)	—	151 (60.9)	38 (56.7)
Any formal education	126 (40.5)	—	97 (39.1)	29 (43.3)
Primigravidae				
No	260 (83.6)	77 (86.5)	213 (85.9)	51 (76.1)
Yes	51 (16.4)	12 (13.5)	35 (14.1)	16 (23.9)
IPTp regime				
MQ full dose	116 (37.3)	27 (30.3)	100 (40.3)	17 (25.4)
MQ split dose	94 (30.2)	27 (30.3)	73 (29.4)	23 (34.3)
SP	101 (32.5)	35 (39.3)	75 (30.2)	27 (40.3)
Malaria at delivery				
No	217 (69.8)	66 (74.2)	175 (70.6)	45 (67.2)
Yes	94 (30.2)	23 (25.8)	73 (29.4)	22 (32.8)
Anaemia at delivery				
No	160 (51.4)	42 (47.2)	131 (52.8)	30 (44.8)
Yes	151 (48.6)	47 (52.8)	117 (47.2)	37 (55.2)
Household possession score				
Mean ± SD	5.4 ± 2.7	—	5.4 ± 2.6	5.6 ± 2.9
HOME				
< 27 (mean)	130 (42.1)	—	99 (40.1%)	33 (50.0)
≥ 27	179 (57.9)	—	148 (59.9%)	33 (50.0)

*Note:* Values are presented as *n* (%) or mean ± SD (Household possession score). — Not applicable: variable not measured or not available for the corresponding group or time point. Continuous variables, including exposure variables (number of episodes of total malaria, symptomatic malaria and malaria with anaemia) were categorized for descriptive presentation in the table, but modelled as continuous variables in regression analyses. *p*‐values were calculated using the original continuous variables (*t*‐test or Wilcoxon rank‐sum test, as appropriate). Categorical variables were compared using Chi‐square or Fisher's exact tests. All analyses were performed using the non‐imputed dataset. * Indicates a difference between included and excluded children (*p* < 0.05). Variables: *Total malaria*, Number of PCR‐ or smear‐positive results from scheduled and emergency visits, recorded from birth to the child's last visit before the 1‐year developmental assessment. *Symptomatic malaria*, Number of positive results with axillary temperature > 37.5°C at the visit or reported within 24 h, recorded from birth to the last visit before the 1‐year developmental assessment. *Malaria with anaemia*, Number of positive results with haemoglobin < 11.0 g/dL, recorded from birth to the last visit before the 1‐year developmental assessment. *ELC*, Early Learning Composite from the Mullen Scales of Early Learning (MSEL), assessed at age 12 months. *GM*, Gross motor scores from MSEL, assessed at age 12 months. *BOT‐2*, Bruininks–Oseretsky Test of Motor Proficiency, Second Edition, assessed at age 6 years. *MPI*, Mental Processing Index from the Kaufman Assessment Battery for Children, Second Edition (KABC‐II), assessed at age 6 years. *NVI*, Non‐verbal Index, assessed at age 6 years. *Preterm birth*, defined as gestational age at delivery < 37 weeks. *Low birthweight*, defined as birth weight < 2500 g. *HAZ*, Height‐for‐age *Z*‐score at age one. *Age at 1‐year assessment*, age (months) at MSEL assessment, conducted at approximately 12 months of age (median 12 [range 12–16]). *Age at 6‐year assessment*, age (months) at BOT‐2 and KABC‐II assessments, conducted at approximately 73 months of age (median 73 [range 70–101]). *Hb at 1‐year assessment*, Haemoglobin concentration (g/dL), measured at the 1‐year developmental assessment. *Hb at 6‐year assessment*, Haemoglobin concentration (g/dL), measured at the 6‐year developmental assessment. *Pre‐MSEL Hb*, Haemoglobin concentration (g/dL), measured at the last visit before the 1‐year developmental assessment. *Number of visits*, Number of healthcare visits (scheduled and emergency) from birth to the last visit before the 1‐year developmental assessment. *Age at the last visit*, Age (months) at the last recorded visit before the 1‐year developmental assessment. *Mother's age*, Age (years) of the mother at inclusion in the MiPPAD study. *IPTp regime*, Type of Intermittent Preventive Treatment in Pregnancy received by the mother during pregnancy, according to the clinical trial protocol: SP = Sulphadoxine‐Pyrimethamine, MQ = Mefloquine. *Malaria at delivery*, Maternal malaria infection at delivery, based on PCR or thick blood smear from peripheral blood. *Anaemia at delivery*, Maternal anaemia status at delivery (haemoglobin < 11.0 g/dL). *Household possession score* (range 0–15), assessed at age 1 year based on ownership of items such as toilets, electricity, television, refrigerator and vehicles. *HOME*, Home Observation for Measurement of the Environment score, assessed at age 1 year through a combination of interview and observation; higher scores indicate more supportive mother–child interactions and learning environments.

In the 1‐year analysis (*n* = 311), children experienced up to six total malaria episodes, four symptomatic episodes and five episodes with anaemia. About half (155/311) had no recorded episodes. In the 6‐year analysis (*n* = 248), children experienced up to five total malaria episodes, three symptomatic malaria episodes and five episodes with anaemia. Approximately half (125/248) had no recorded episodes. In both analyses, 60% of children attended at least six visits, including both scheduled and unscheduled visits (median: 6; IQR‐1‐year: 5–7; IQR‐6‐year: 5–8).

### 3.2 Unadjusted Associations Between Malaria and Neurodevelopment

Children with a higher number of malaria episodes tended to have lower motor scores, whereas cognitive scores showed no consistent patterns (Table 2). In unadjusted models (Supporting Information S3), at age one, total malaria (*β*: −1.58; [−2.97, −0.20]), symptomatic malaria (*β*: −3.19; [−5.13, −1.26]) and malaria with anaemia (*β*: −1.94 [−3.41, −0.47]) were each associated with lower motor scores. Total malaria (*β*: −1.50; [−2.89, −0.12]), and malaria with anaemia (*β*: −1.71 [−3.18, −0.23]) were also associated with lower cognitive scores at age one. At age six, only symptomatic malaria was associated with lower motor scores (*β*: −1.53; [−2.93, −0.13]).

**TABLE 2:** Bivariate analysis of factors associated with neurodevelopmental outcomes at ages 1 (GM and ELC, *N* = 311) and 6 years (BOT‐2, MPI and NVI, *N* = 248).

	GM	ELC	BOT‐2	MPI	NVI
All	51.4 ± 14.3	99.6 ± 14.3	39.1 ± 9.0	59.7 ± 11.9	52.0 ± 7.8
Total malaria	*	*	*		
0	52.5 ± 14.9	100.9 ± 14.5	40.0 ± 8.9	60.6 ± 12.2	51.7 ± 7.8
1	52.9 ± 13.7	101.1 ± 13.2	39.1 ± 9.6	58.8 ± 12.1	51.5 ± 7.7
2	50.7 ± 13.4	95.8 ± 15.9	38.2 ± 9.6	58.5 ± 12.2	53.0 ± 8.4
≥ 3	45.4 ± 13.0	96.1 ± 13.1	36.3 ± 7.4	59.1 ± 10.0	52.6 ± 7.6
Symptomatic malaria	***		*		
0	53.1 ± 14.8	100.9 ± 14.2	40.0 ± 9.1	60.7 ± 12.3	51.7 ± 7.9
1	50.7 ± 13.2	98.5 ± 14.5	38.3 ± 9.2	58.3 ± 11.9	52.4 ± 7.8
2	47.2 ± 13.4	94.5 ± 14.1	36.0 ± 8.5	56.6 ± 9.0	51.6 ± 6.7
≥ 3	40.9 ± 10.8	102.3 ± 13.6	38.8 ± 5.8	64.3 ± 12.1	55.7 ± 9.9
Malaria with anaemia	*	*	*		
0	53.0 ± 15.0	101.1 ± 14.4	39.7 ± 8.8	60.4 ± 11.8	51.7 ± 7.6
1	51.0 ± 12.4	100.1 ± 13.0	39.9 ± 9.7	58.1 ± 12.4	51.2 ± 7.6
2	49.7 ± 14.1	93.9 ± 16.1	37 ± 10.6	59.5 ± 13.3	54.8 ± 8.7
≥ 3	45.4 ± 13.0	97.0 ± 13.4	36.0 ± 5.9	59.8 ± 9.5	52.0 ± 7.8
Sex		*	***		*
Male	52.0 ± 14.5	97.5 ± 13.8	41.9 ± 8.1	60.5 ± 12.4	52.3 ± 7.9
Female	51.0 ± 14.2	101.7 ± 14.6	36.5 ± 9.1	59.0 ± 11.0	51.6 ± 7.7
Preterm birth	*		*		
No	51.2 ± 14.2	99.9 ± 14.0	39.2 ± 9.0	60.0 ± 11.8	52.0 ± 7.8
Yes	55.3 ± 15.5	96.3 ± 18.7	37.6 ± 9.3	55.2 ± 12.7	50.7 ± 7.9
Low birthweight					
No	51.6 ± 14.2	99.7 ± 14.3	39.4 ± 9.0	59.7 ± 11.8	51.9 ± 7.8
Yes	49.8 ± 15.8	98.5 ± 16.1	36.3 ± 9.8	60.5 ± 13.8	51.4 ± 7.2
HAZ	***		***		
< −2 (Stunting)	46.0 ± 13.1	99.9 ± 16.2	35.5 ± 9.9	58.9 ± 11.4	51.2 ± 6.9
≥ −2	53.1 ± 14.3	99.7 ± 13.9	40.1 ± 8.5	60.0 ± 12.4	52.3 ± 8.0
Age at 1‐year assessment (month)	*	***			
< 13 (mean)	53.5 ± 14.2	102.8 ± 13.7	—	—	—
≥ 13	49.3 ± 14.2	96.4 ± 14.4	—	—	—
Age at 6‐year assessment (month)			***	*	***
< 73 (median)	—	—	38.1 ± 7.9	60.9 ± 40.5	52.1 ± 7.6
≥ 73	—	—	39.5 ± 9.5	59.2 ± 12.4	51.9 ± 7.9
Hb at 1‐year assessment (g/dL)					
< 11.0 (anaemic)	99.8 ± 14.8	50.6 ± 14.0	—	—	—
≥ 11.0	99.4 ± 13.4	53.6 ± 14.9	—	—	—
Hb at 6‐year assessment (g/dL)			*		
< 11.0 (anaemic)	—	—	36.1 ± 7.5	58.6 ± 12.3	52.9 ± 7.9
≥ 11.0	—	—	39.1 ± 8.7	60.4 ± 11.6	52.4 ± 7.7
Pre‐MSEL Hb	*		*		
< 11.0 (anaemic)	51.0 ± 14.1	99.9 ± 14.7	38.8 ± 9.1	59.6 ± 12.4	51.6 ± 7.8
≥ 11.0	52.3 ± 14.7	99.2 ± 13.8	39.6 ± 9.0	60.0 ± 11.0	52.7 ± 7.8
Number of visits					
< 6 (median)	51.8 ± 15.0	99.6 ± 13.7	38.9 ± 8.2	59.6 ± 11.1	52.0 ± 7.1
≥ 6	51.2 ± 13.9	99.7 ± 14.8	39.2 ± 9.5	59.8 ± 12.4	51.9 ± 8.2
Age at the last visit (month)					
< 12 (median)	51.4 ± 15.7	101.0 ± 16.3	41.3 ± 10.6	61.9 ± 11.6	52.9 ± 9.0
≥ 12	51.5 ± 14.2	99.5 ± 14.1	38.8 ± 8.8	59.4 ± 11.9	51.8 ± 7.6
Mother's age (year)			*	*	
< 25	50.8 ± 13.6	101.0 ± 13.3	38.4 ± 8.2	58.8 ± 12.7	51.3 ± 7.2
≥ 25	52.0 ± 13.6	98.7 ± 15.0	39.5 ± 9.5	60.3 ± 11.4	52.4 ± 8.1
Mother's education		***		*	
None	50.4 ± 13.4	96.5 ± 14.4	38.3 ± 9.0	58.1 ± 12.3	52.0 ± 8.1
Any formal education	53.0 ± 15.5	104.2 ± 13.0	40.3 ± 9.0	62.0 ± 10.7	51.9 ± 7.3
Primigravidae	*				
No	52.3 ± 13.7	99.3 ± 14.4	39.3 ± 9.1	59.4 ± 11.2	52.0 ± 7.8
Yes	47.1 ± 16.6	101.3 ± 13.9	37.6 ± 8.7	61.4 ± 15.7	51.9 ± 8.0
IPTp regime					
MQ full dose	50.2 ± 13.6	98 ± 13.9	38.6 ± 9.0	58.6 ± 10.5	52.1 ± 7.6
MQ split dose	51.1 ± 15.8	99.4 ± 13.5	40.2 ± 10	61.9 ± 14.1	52.9 ± 7.7
SP	53.3 ± 13.7	101.8 ± 15.4	38.6 ± 8.3	59.0 ± 11.2	50.9 ± 8.1
Malaria at delivery					
No	52.2 ± 14.2	99.6 ± 14.6	39.7 ± 8.9	60.5 ± 12.1	51.9 ± 7.2
Yes	49.8 ± 14.5	99.8 ± 13.9	37.5 ± 9.3	57.9 ± 11.2	52.0 ± 9.1
Anaemia at delivery					
No	51.4 ± 14.5	100.0 ± 14.0	39.2 ± 9.0	60.5 ± 11.5	52.8 ± 8.1
Yes	51.6 ± 14.2	99.3 ± 14.8	39.0 ± 9.2	58.9 ± 12.3	51.0 ± 7.4
Household possession score	*	***	*	***	
Low (1–4; tercile)	49.3 ± 13.7	96.9 ± 15.3	37.5 ± 8.0	58.3 ± 10.9	51.7 ± 7.7
Medium (5–6)	50.4 ± 14.7	99.1 ± 14.3	38.9 ± 10.1	58.5 ± 13.5	51.5 ± 8.3
High (7–13)	54.6 ± 14.1	102.8 ± 13.0	40.8 ± 8.7	62.2 ± 10.9	52.8 ± 7.4
HOME		***		*	
< 27 (mean)	50.1 ± 13.4	97.6 ± 15.5	39.1 ± 8.5	57.8 ± 10.8	51.5 ± 7.9
≥ 27	52.4 ± 14.9	101.1 ± 13.4	39.1 ± 9.4	61.0 ± 12.5	52.3 ± 7.7

*Note:* Values are presented as mean ± SD. — Not applicable: variable not measured or not available for the corresponding group or time point. Continuous variables, including exposure variables (number of episodes of total malaria, symptomatic malaria and malaria with anaemia) were categorized for descriptive presentation in the table, but modelled as continuous variables in regression analyses. *p*‐values were derived from unadjusted generalized linear models (GLMs) with a Gaussian distribution and identity link function, using the non‐imputed dataset. *p < 0.05, **p < 0.01, ***p < 0.001. Variables: *Total malaria*, Number of PCR‐ or smear‐positive results from scheduled and emergency visits, recorded from birth to the child's last visit before the 1‐year developmental assessment. *Symptomatic malaria*, Number of positive results with axillary temperature > 37.5°C at the visit or reported within 24 h, recorded from birth to the last visit before the 1‐year developmental assessment. *Malaria with anaemia*, Number of positive results with haemoglobin < 11.0 g/dL, recorded from birth to the last visit before the 1‐year developmental assessment. *ELC*, Early Learning Composite from the Mullen Scales of Early Learning (MSEL), assessed at age 12 months. *GM*, Gross motor scores from MSEL, assessed at age 12 months. *BOT‐2*, Bruininks–Oseretsky Test of Motor Proficiency, Second Edition, assessed at age 6 years. *MPI*, Mental Processing Index from the Kaufman Assessment Battery for Children, Second Edition (KABC‐II), assessed at age 6 years. *NVI*, Non‐verbal Index, assessed at age 6 years. *Preterm birth*, defined as gestational age at delivery < 37 weeks. *Low birthweight*, defined as birth weight < 2500 g. *HAZ*, Height‐for‐age *Z*‐score at age one. *Age at 1‐year assessment*, age (months) at MSEL assessment, conducted at approximately 12 months of age (median 12 [range 12–16]). *Age at 6‐year assessment*, age (months) at BOT‐2 and KABC‐II assessments, conducted at approximately 73 months of age (median 73 [range 70–101]). *Hb at 1‐year assessment*, Haemoglobin concentration (g/dL), measured at the 1‐year developmental assessment. *Hb at 6‐year assessment*, Haemoglobin concentration (g/dL), measured at the 6‐year developmental assessment. *Pre‐MSEL Hb*, Haemoglobin concentration (g/dL), measured at the last visit before the 1‐year developmental assessment. *Number of visits*, Number of healthcare visits (scheduled and emergency) from birth to the last visit before the 1‐year developmental assessment. *Age at the last visit*, Age (months) at the last recorded visit before the 1‐year developmental assessment. *Mother's age*, Age (years) of the mother at inclusion in the MiPPAD study. *IPTp regime*, Type of Intermittent Preventive Treatment in Pregnancy received by the mother during pregnancy, according to the clinical trial protocol: SP = Sulphadoxine‐Pyrimethamine, MQ = Mefloquine. *Malaria at delivery*, Maternal malaria infection at delivery, based on PCR or thick blood smear from peripheral blood. *Anaemia at delivery*, Maternal anaemia status at delivery (haemoglobin < 11.0 g/dL). *Household possession score* (range 0–15), assessed at age 1 year based on ownership of items such as toilets, electricity, television, refrigerator and vehicles. *HOME*, Home Observation for Measurement of the Environment score, assessed at age 1 year through a combination of interview and observation; higher scores indicate more supportive mother–child interactions and learning environments.

In bivariate analyses, preterm birth (gestational age < 37 weeks [31]), HAZ and Hb at the last visit before MSEL‐assessment were associated with motor scores at both ages. HOME score and maternal education were associated with cognitive scores at both ages, while maternal age and Hb at 6‐year assessment were associated with cognitive scores only at 6 years. Household possession score was associated with motor and cognitive outcomes at both ages. Sex was associated with 1‐year cognitive scores and 6‐year motor scores. Low birthweight (birthweight < 2500 g [32]), Hb at 1‐year assessment, maternal malaria or anaemia at delivery and maternal IPTp regimen were not associated with neurodevelopmental outcomes.

### 3.3 Adjusted Associations Between Malaria and Neurodevelopment

In adjusted models (Figure 2), early‐life malaria was associated with lower motor scores at both ages. At age one, a higher number of symptomatic malaria episodes (*β*: −2.81 [−4.73, −0.89]) and malaria with anaemia (*β*: −1.57 [−3.04, −0.10]) were associated with lower motor scores. At age six, associations were observed for total malaria (*β*: −0.93 [−1.80, −0.07]), symptomatic malaria (*β*: −1.43 [−2.65, −0.22]) and malaria with anaemia (*β*: −0.96 [−1.86, −0.05]). Although some confidence intervals approached zero, indicating limited precision, associations consistently suggested lower motor scores. No malaria exposures were associated with cognitive outcomes at 1 (Supporting Information S4) or 6 years.

**FIGURE 2:**
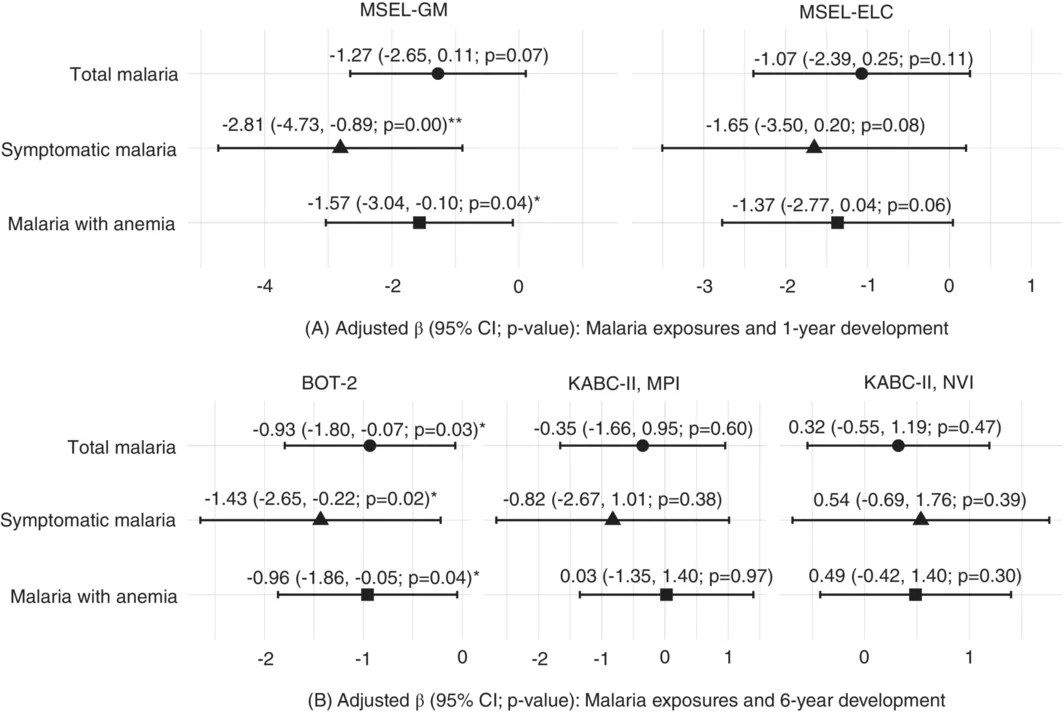
Adjusted associations between early‐life malaria exposures from birth to 12 months and developmental outcomes at ages 1 and 6 years. Panel A shows outcomes assessed at 1 year, and Panel B shows outcomes assessed at 6 years. Estimates were obtained from multivariable generalized linear models with a Gaussian distribution and identity link. Malaria exposures were defined as number of episodes recorded from birth up to each child's last visit before the 1‐year developmental assessment and include: Total malaria (solid circle; PCR or thick blood smear positive), symptomatic malaria (solid triangle; PCR or thick blood smear positive with an axillary temperature > 37.5°C at the visit or reported within the previous 24 h) and malaria with anaemia (solid square; PCR or thick blood smear positive with haemoglobin < 11.0 g/dL). In the regression models, malaria episodes were modelled as continuous variables. Developmental outcomes at age 1 year include the Early Learning Composite (ELC) and Gross Motor (GM) scores from the Mullen Scales of Early Learning (MSEL). Outcomes at age 6 years include the Bruininks‐Oseretsky Test of Motor Proficiency, Second Edition (BOT‐2), and cognitive outcomes assessed using the Kaufman Assessment Battery for Children, Second Edition (KABC‐II), including the Mental Processing Index (MPI) and Non‐Verbal Index (NVI). All models were adjusted for child sex, age at developmental assessment, height‐for‐age *Z*‐score (HAZ) at 1 year, maternal IPTp regime, maternal malaria at delivery, primigravida status, maternal education and HOME and Household possession scores. *p < 0.05, **p < 0.01, ***p < 0.001.

In sensitivity analysis, estimates remained similar after additional adjustment for concurrent Hb and after excluding PCR‐positive but TBS negative episodes. When extending the exposure window to 24 months, only a limited number of additional episodes were captured (16 additional total malaria, 2 symptomatic episodes and 5 malaria with anaemia) and associations with 6‐year outcomes remained similar (Supporting Information S5).

### 3.4 Mediation Analysis

Mediation analysis suggested partial mediation by Hb at the last visit before MSEL‐assessment on the association between symptomatic malaria and motor scores. At age one, ACME was −0.31 (95% CI: −0.80, 0.00, *p* = 0.04), corresponding to 11% of the total effect. At age six ACME was −0.23 (95% CI: −0.53, 0.00, *p* = 0.047), corresponding to 16% of the total effect. Although some confidence intervals approached zero, indicating limited precision, the ACME estimates consistently suggested a mediation effect. Sensitivity analysis showed no mediation with alternative Hb measures.

## 4 Discussion

The results suggest that a higher number of uncomplicated malaria episodes during infancy, particularly symptomatic malaria and malaria with anaemia, are associated with poorer motor development at ages one and six, partially and potentially mediated by lower haemoglobin concentration. No associations were observed between early‐life malaria and cognitive outcomes at either age.

These findings partially align with a previous study in Benin showing that malaria in pregnancy (MiP) was associated with poorer motor outcomes at 1 year and cognitive outcomes at 6 years [6]. Similarly, Weckman et al. [7] reported that MiP was linked to poorer language development at age two in Malawian children. These different patterns suggest that the timing of malaria exposure may influence different neurodevelopmental domains.

Evidence on infancy malaria is mixed. Boivin et al. [5] found that infancy malaria was associated with lower cognitive scores at ages two and three in Ugandan children, while Bangirana et al. [8] observed cognitive deficits at 24 months but not at 12 or 36 months. Bangirana et al. [9] also found that children under five with cerebral malaria had cognitive impairment up to 1 year after hospital discharge. Recent evidence shows long‐term cognitive impairment following cerebral malaria and severe malarial anaemia [14]. Our interpretation is that associations between malaria and neurodevelopment may vary by infection severity and timing, reflecting differences in brain maturation, which may explain the absence of cognitive associations in our study.

In our study, total malaria was associated with motor outcomes only at 6 years, while symptomatic malaria and malaria with anaemia were associated with motor outcomes at both ages. This difference may reflect the definition of total malaria, which includes both symptomatic and asymptomatic infections. Many total malaria cases among children in the 1‐year analysis were likely asymptomatic, causing only subtle effects. Although there were no major differences between children included and excluded from the analyses, symptomatic children may have been more likely to seek medical care and participate in the study, potentially resulting in a higher proportion of symptomatic cases in the 6‐year analysis. Some associations had confidence intervals close to zero, reflecting the relatively small number of children who experienced malaria (about half had at least one episode), variability in exposures and natural fluctuations in motor scores. However, the overall pattern consistently suggests that early‐life malaria is linked to poorer motor development. In the sensitivity analysis extending the exposure window to 24 months, associations with 6‐year outcomes remained largely unchanged. This likely reflects the limited number of additional malaria episodes occurring after infancy in this cohort, suggesting that malaria exposure during infancy captured the majority of childhood malaria burden.

Mediation analyses suggested that lower Hb at the last visit before MSEL‐assessment may partially and potentially mediate the associations between uncomplicated malaria and motor development at both ages. Mediation effects had confidence intervals approaching zero, reflecting differences in timing between Hb measurement and the MSEL‐assessment among children, natural fluctuations in Hb and motor scores, or recovery from earlier anaemia. Because mediation only captures part of the total effect, the estimates can be small. However, the findings support a role for Hb in linking malaria to motor development. Consistent with this, Olney et al. [13] reported cross‐sectional associations between malaria, lower Hb and reduced motor development and activities in children aged 10–14 months. Sensitivity analyses using alternative Hb measures showed no evidence of mediation, suggesting that Hb measured closer to infection is more relevant for short‐term motor outcomes, and that recovery from earlier anaemia may reduce longer‐term impacts. Therefore, incorporating routine anaemia screening to malaria programmes is important for timely treatment and reducing developmental consequences.

These findings are consistent with known biological and behavioural mechanisms. Severe and cerebral malaria trigger strong neuroinflammatory responses, including altered cytokine and chemokine profiles, dysregulated regulatory T cells and changes in lipid mediators. Sequestration of infected erythrocytes, local hypoxemia, ischemia and oxidative stress can impair the blood–brain barrier, allowing inflammatory mediators, reactive oxygen species, CD8+ T cells and parasite toxins to enter the central nervous system [33, 34]. While repeated uncomplicated malaria is unlikely to induce such intense responses, milder, recurrent infections may cumulatively disrupt motor circuits. Uncomplicated malaria can also cause anaemia through haemolysis, impaired red blood cell production and inflammation, lowering Hb and reducing cerebral oxygen delivery, thereby affecting high‐energy motor process (e.g., myelination, synaptic function and neuromuscular coordination) [11, 35]. These effects may be particularly relevant during early childhood, when motor systems develop rapidly, while cognitive functions mature later [2, 3, 14]. Illness‐related fatigue may limit engagement in age‐appropriate activities (e.g., crawling, walking and playing), and increased caregiver assistance restrict opportunities for independent motor practice [13]. Collectively, these mechanisms provide a coherent explanation for the observed associations between early‐life malaria and motor development, which relatively modest in borderline confidence intervals, and for the absence of associations with cognitive outcomes at early and middle childhood.

To our knowledge, this is the first study to examine the association between uncomplicated malaria infections in infancy and neurodevelopmental outcomes beyond early childhood, using data from a prospective birth cohort nested within a well‐characterized clinical trial. Children were followed from the prenatal period through mid‐childhood, enabling adjustment for a broad range of potential confounders. Malaria exposure was measured longitudinally using both molecular and microscopic methods, allowing detailed characterization of infection burden, while neurodevelopmental outcomes were assessed using standardized tools validated for this study setting. Assessment at both 1 and 6 years allowed examination of short‐ and longer‐term neurodevelopmental patterns, and the consistency of findings across sensitivity analyses supports the robustness of our results.

Several limitations should be considered. First, asymptomatic or mild infections may have been missed as unscheduled visits tested only symptomatic children, potentially leading to non‐differential misclassification and attenuated associations. Second, the independent effects of asymptomatic parasitaemia could not be isolated. Third, residual confounding is possible, as anaemia may have causes other than malaria. Fourth, factors between 24 months and 6 years were not included to avoid over‐adjustment, and associations may partly reflect later exposures. Koura et al. [28] reported excellent interrater reliability for the MSEL in this setting, but reliability of the KABC‐II and BOT‐2 was not specifically assessed in our cohort. In addition, neurodevelopmental assessments lacked Beninese norms, limiting comparisons with external populations. Model assumptions of linearity, moderate sample size and uneven exposure distribution may also limit precision and generalizability.

Future research in larger cohorts and diverse settings is needed to improve statistical power and generalizability and to develop and use locally normed neurodevelopmental assessments. Studies should identify sensitive developmental periods when malaria infection most affects neurodevelopmental domains. Incorporating immunological biomarkers could clarify biological pathways linking infection, immunity and neurodevelopment. Longer‐term follow‐up into adolescence would determine whether early deficits persist, resolve, or affect broader functional outcomes.

## 5 Conclusion

A higher number of uncomplicated malaria episodes during infancy was associated with poorer motor development that may persist into mid‐childhood, partially and potentially mediated by lower haemoglobin concentration. These findings show that uncomplicated malaria is not a benign condition and pose an important risk to child development. Strengthening malaria prevention and anaemia management within routine child health services may improve developmental outcomes and support long‐term human capital development.

## Funding

This work was supported by the Fondation de France [grant number 2015 00060746 to F.B.‐L.]; the Eunice Kennedy Shriver National Institute of Child Health and Human Development [grant number NIH/NICHD‐R21‐HD060524 to F.B.‐L.] and the European and Developing Countries Clinical Trials Partnership [grant number EDCTP‐IP.07.31080.002 to M.C.].

## Conflicts of Interest

The authors declare no conflicts of interest.

## Supporting information


**Section S1**: Overview of follow‐up and data collection in mothers (pregnancy to delivery) and APEC children (birth to 24 months and at 6 years).
**Section S2**: Directed acyclic graph (DAG) representing the hypothesized causal relationships between early‐life malaria and neurodevelopment.
**Section S3**: Unadjusted Associations between early‐life malaria exposures from birth to 12 months and developmental outcomes at ages 1 and 6 years.
**Section S4**: Adjusted associations between early‐life malaria exposures from birth to 12 months and MSEL ELC subscores.
**Section S5**: Adjusted association between early‐life malaria exposures from birth to 24 months and developmental outcome at age six.

## Data Availability

The data underlying this article cannot be shared publicly due to privacy concerns regarding the individuals who participated in the study.
