# Supplementary material for: Uncomplicated Malaria in Infancy and Neurodevelopment at 1 and 6 Years: Evidence From the MiPPAD Birth Cohort Study of Benin
Source: Trop Med Int Health. 2026 Jun 19;31(10):1395–407. doi: 10.1111/tmi.70179 (PMC13628417; doi:10.1111/tmi.70179)
Supplement: Supplementary file 1 [file TMI-31-1395-s001.docx]

**Supplementary Material: Uncomplicated Malaria in Infancy and Neurodevelopment at One and Six Years: Evidence from the MiPPAD Birth Cohort in Benin**

**Table of Contents**

| **Section** | **Title** | **Page** |
| --- | --- | --- |
| **S1** | Overview of data collection in mothers and children from pregnancy up to 24 months and at age six years | 1 |
| **S2** | Directed acyclic graph (DAG) representing the hypothesized causal relationships between early-life malaria and neurodevelopment. | 2 |
| **S3** | Unadjusted Associations between early-life malaria exposures from birth to 12 months and developmental outcomes at ages one and six years | 3 |
| **S4** | Adjusted associations between early-life malaria exposures from birth to 12 months and MSEL ELC subscores. | 4 |
| **S5** | Adjusted association between early-life malaria exposures from birth to 24 months and developmental outcome at age six. | 5 |

**S1: Overview of follow-up and data collection in mothers (pregnancy to delivery) and APEC children (birth to 24 months and at 6 years)**

*Based on : Accrombessi M, Ouédraogo S, Agbota GC, Gonzalez R, Massougbodji A, Menéndez C, et al. Malaria in pregnancy is a predictor of infant haemoglobin concentrations during the first year of life in Benin, West Africa. PLoS ONE. 2015;10(6):e0129510.*

** only if participants presented with malaria clinical signs*

***Hb****, Hemoglobin concentration;* ***TBS****, Thick blood smear;* ***HOME,*** *Home Observation for Measurement of the Environment inventory (HOME), derived from maternal interviews and observed mother–child interactions during home visit, with higher scores reflecting a more supportive environment.;* ***Household asset score*** *(range 0-15) based on electricity, television, toilet, refrigerator, livestock, and vehicles.;* ***MSEL****, Mullen Scales of Early Learning;* ***BOT-2****, Bruininks–Oseretsky Test of Motor Proficiency, Second Edition;* ***KABC-II****, Kaufman Assessment Battery for Children, Second Edition*

**S2. Directed acyclic graph (DAG) representing the hypothesized causal relationships between early-life malaria and neurodevelopment.**

The DAG illustrates the hypothesized relationships between early-life malaria (exposure), neurodevelopment (outcome), haemoglobin concentration (Hb), and covariates included in the analysis, based on prior literature and biological plausibility. Early-life malaria refers to the number of malaria episodes occurring from birth to 12 months of age and was defined using three measures: total malaria (PCR or thick blood smear positive), symptomatic malaria (PCR or thick blood smear positive with an axillary temperature >37.5°C at the visit or reported within the previous 24 hours), and malaria with anaemia (PCR or thick blood smear positive with haemoglobin <11.0 g/dL).

The same causal structure was used for both neurodevelopmental assessments at 1 year and 6 years of age; the outcome node represents neurodevelopment measured at the corresponding time point. Developmental outcomes at age 1 year include the Early Learning Composite (ELC) and Gross Motor (GM) scores from the Mullen Scales of Early Learning (MSEL).Outcomes at age 6 years include the Bruininks-Oseretsky Test of Motor Proficiency, Second Edition (BOT-2), and cognitive outcomes assessed using the Kaufman Assessment Battery for Children, Second Edition (KABC-II), including the Mental Processing Index (MPI) and Non-Verbal Index (NVI).

**Variables:**

**Hb**, Haemoglobin concentration (g/dL), measured at the last visit before the 1-year developmental assessment

**IPTp**, Type of Intermittent Preventive Treatment in Pregnancy received by the mother during pregnancy, according to the clinical trial protocol: SP = Sulfadoxine-Pyrimethamine, MQ = Mefloquine

**Malaria at delivery**, Maternal malaria infection at delivery, based on PCR or thick blood smear from peripheral blood

**Household asset score** (range 0-15), assessed at age one year based on ownership of items such as toilets, electricity, television, refrigerator, and vehicles

**HOME**, Home Observation for Measurement of the Environment score, assessed at age one year through a combination of interview and observation; higher scores indicate more supportive mother–child interactions and learning environments

**HAZ**, Height-for-age Z-score at age one

**Age**, age (months) at neurodevelopmental assessment

**S3: Unadjusted Associations between early-life malaria exposures from birth to 12 months and developmental outcomes at ages one and six years**

Panel A shows outcomes assessed at one year, and Panel B shows outcomes assessed at six years. Estimates were obtained from unadjusted generalized linear models with a Gaussian distribution and identity link. Malaria exposures were defined as number of episodes recorded from birth up to each child’s last visit before the 1-year developmental assessment and include: total malaria (solid circle; PCR or thick blood smear positive), symptomatic malaria (solid triangle; PCR or thick blood smear positive with an axillary temperature >37.5°C at the visit or reported within the previous 24 hours), and malaria with anaemia (solid square; PCR or thick blood smear positive with haemoglobin <11.0 g/dL). In the regression models, malaria episodes were modelled as continuous variables. Developmental outcomes at age 1 year include the Early Learning Composite (ELC) and Gross Motor (GM) scores from the Mullen Scales of Early Learning (MSEL).Outcomes at age 6 years include the Bruininks-Oseretsky Test of Motor Proficiency, Second Edition (BOT-2), and cognitive outcomes assessed using the Kaufman Assessment Battery for Children, Second Edition (KABC-II), including the Mental Processing Index (MPI) and Non-Verbal Index (NVI). *p < 0.05, **p < 0.01, ***p < 0.001.

**S4: Adjusted associations between early-life malaria exposures from birth to 12 months and MSEL ELC subscores.**

| **Exposures** | **Visual perception** | **Fine motor skills** | **Receptive Language** | **Expressive Language** |
| --- | --- | --- | --- | --- |
| **Total malaria** | -0.90 (-1.96,0.17) | -0.97 (-1.92,0.01) | -0.25 (-0.87,0.38) | -0.17 (-1.14,0.80) |
| **Symptomatic malaria** | -0.98 (-2.47,0.52) | -1.10 (-2.42,0.27) | -0.53 (-1.41,0.35) | -0.98 (-2.34,0.38) |
| **Malaria with anaemia** | -1.17 (-2.30,0.00) | -1.05 (-2.07,0.03) | -0.35 (-1.02,0.32) | -0.32 (-1.35,0.71) |

*Values are β coefficients (95% confidence intervals) from multivariable generalized linear models with a Gaussian distribution and identity link. Malaria exposures included total malaria (PCR- or thick blood smear-positive), symptomatic malaria (PCR- or thick blood smear-positive with axillary temperature >37.5°C or reported fever within the previous 24 hours), and malaria with anaemia (PCR- or thick blood smear-positive with haemoglobin <11.0 g/dL), recorded from birth to the last visit before the 1-year assessment. Malaria episodes were modelled as continuous variables. Models were adjusted for child sex, age at assessment, one-year HAZ, IPTp, maternal malaria at delivery, primigravida status, maternal education, and HOME and household possession scores.*

**S5: Adjusted association between early-life malaria exposures from birth to 24 months and developmental outcome at age six.**

Estimates were obtained from multivariable generalized linear models with a Gaussian distribution and identity link. Malaria exposures were defined as number of episodes recorded from birth up to each child’s last visit before the 1-year developmental assessment and include: total malaria (solid circle; PCR or thick blood smear positive), symptomatic malaria (solid triangle; PCR or thick blood smear positive with an axillary temperature >37.5°C at the visit or reported within the previous 24 hours), and malaria with anemia (solid square; PCR or thick blood smear positive with hemoglobin <11.0 g/dL). In the regression models, malaria episodes were modelled as continuous variables. Developmental outcomes at age 6 include the Bruininks-Oseretsky Test of Motor Proficiency, Second Edition (BOT-2), and cognitive outcomes assessed using the Kaufman Assessment Battery for Children, Second Edition (KABC-II), including the Mental Processing Index (MPI) and Non-Verbal Index (NVI). All models were adjusted for child sex, age at developmental assessment, height-for-age Z-score (HAZ) at one-year, IPTp, maternal malaria at delivery, primigravida status, maternal education, and HOME and Household possession scores. *p < 0.05, **p < 0.01, ***p < 0.001
